# Numerical Analysis of the Aerodynamic Interactions in Tandem Flying Snake Airfoils

**DOI:** 10.3390/biomimetics10030174

**Published:** 2025-03-12

**Authors:** Yuchen Gong, Jiacheng Guo, Alexander He, Ye Sun, Haibo Dong

**Affiliations:** 1 Department of Mechanical and Aerospace Engineering, University of Virginia, Charlottesville, VA 22903, USA; yg6nc@virginia.edu (Y.G.); mns4zh@virginia.edu (J.G.); dzv7sg@virginia.edu (Y.S.); 2 Department of Aerospace Engineering, University of Maryland, College Park, MD 20740, USA; alex1216@terpmail.umd.edu

**Keywords:** flying snake gliding, tandem airfoils, computational fluid dynamics, vortex dynamics

## Abstract

During gliding, flying snakes flatten their ribs to create an airfoil-like cross-section and adopt S-shape postures, allowing upstream body segments to generate wake structures that affect the aerodynamic performance of downstream segments. This study investigates these interactions using numerical simulations of two-dimensional snake cross-sectional airfoils. By employing an immersed-boundary-method-based incompressible flow solver with tree topological local mesh refinement, various foil positions and movements were analyzed. The results show that aligning the downstream foil with the upstream foil reduces lift production by 86.5% and drag by 96.3%, leading to a 3.77-fold increase in the lift-to-drag ratio compared to a single airfoil. This improvement is attributed to the vortex–wedge interaction between the upstream vortex and the following foil’s leading edge (wedge), which enhances the gliding efficiency of the posterior body. Furthermore, integrating specific pitching motions with coordinated vortex shedding could further optimize its lift production. These findings provide valuable insights into the aerodynamics of tandem flying snake airfoils, offering guidance for configuring optimal body postures for improving gliding efficiency.

## 1. Introduction

In the study of animal flight and fish swimming, we often observe biologically structured two-foil systems, such as pairs of insect wings and sets of fins on fish bodies. Evidence indicates that such systems gain aerodynamic or hydrodynamic advantages from the vortex interactions between the upstream and downstream foils. Warkentin et al. [1] conducted experimental work that studied the aerodynamics of two insect wings with different spacing, flapping frequencies, and phase angles. This study provided foundational guidance for a systematic parametric investigation of a two-foil system. Wang et al. numerically studied passively pitching tandem plates inspired by insect hovering flight. It was found that the wing–wing interaction is essential for wake capturing and the aerodynamic performance of the tandem foils [2,3]. Tandem foil systems in aquatic creatures have been extensively studied, with research demonstrating significant improvements in their hydrodynamic performance. For example, in a dorsal fin–caudal fin system of a swimming fish, experiments have shown that the caudal fin, located downstream of the dorsal fin, interacts with the vortices shed from the upstream in different species of fish [4,5,6]. Canonical tandem hydrofoil experiments have also revealed the importance of active pitching motion in the flow interactions between multiple tandem foils and the resulting collective force generation [7,8,9,10,11], finding that the downstream foils are strongly affected by the spacing and the phase difference between the foil pitching motion and the wake vortices. Such performance enhancement can also be observed with larger objects. A study by Beal et al. found that, even outside the suction region of a bluff cylinder, a deceased fish can be propelled upstream if its flexible body resonates with the oncoming vortices generated in the wake of the cylinder [12]. Many studies of the flight of insects and biomimetic flapping wings also discovered the importance of tandem wing configurations in lift generation [2,3]. A study on fluid–structure interactions in hovering flight [13] provided relevant insights into the role of passive deformation in unsteady flows, which can inform the analysis of foil–foil interactions in flying snake gliding.

**Figure 1:**
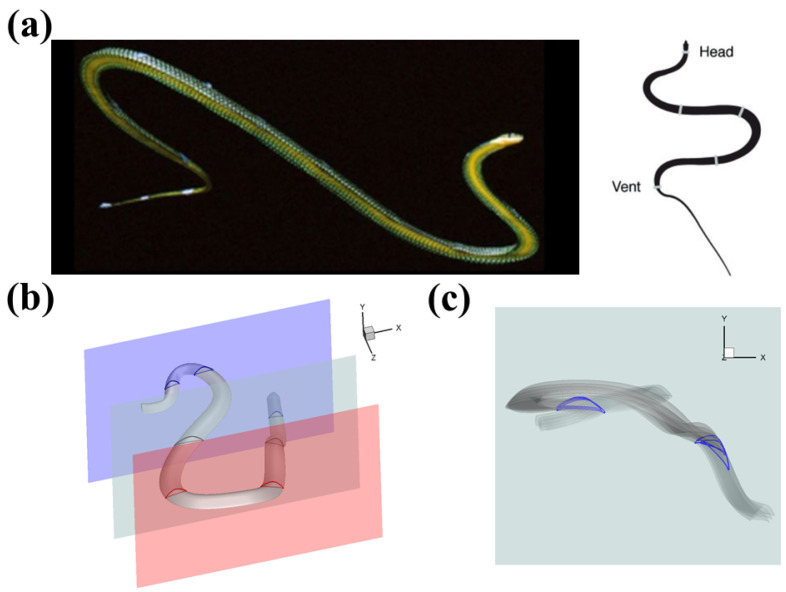
(**a**) The snake applies undulating motion and forms an “S” shape in the aerial gliding [15]. During gliding, their anterior and posterior body create the tandem configuration [25]. (**b**) Perspective view of the 3D flying snake model with several slice cuts showing different tandem snake airfoil configurations. (**c**) Side view of the flying snake motion during gliding, indicating the pitching angle change in the posterior body.

During aerial gliding, flying snakes (*Chrysopelea*) tend to adopt a foil–foil interaction configuration [14,15,16,17,18]. As a highly specialized gliding species, these snakes utilize a unique form of locomotion that involves bending their bodies to create a large, undulating motion (Figure 1a). The curved posture forms an S shape, allowing the anterior and posterior sections of the snake’s body to align in a tandem formation (Figure 1b). Miklasz et al. were the first to discuss this formation and studied the interaction between the bodies using two in-line cylindrical foils [19]. Their research revealed that the lift on the downstream foil is affected by the vertical displacement between the anterior and posterior sections of the snake. Subsequently, a more detailed analysis of the snake’s cross-sectional shape was conducted, both experimentally [20] and numerically [21]. The cross-section of the snake body is shaped like a triangle, featuring a flat ventral surface and rounded edges at both ends (see Figure 1). This foil shape has been shown to play a significant role in generating lift. Based on this shape, Jafari et al. [22] further investigated the interactions between the tandem body segments. Their study examined a wide range of horizontal (“the gap”) and vertical (“the staggered”) distances. Additionally, they explored different combinations of the orientation (the angle of attack (*AOA*)) between the two airfoils. The results revealed that the average lift-to-drag ratio reached a maximum value of 2.2, which was almost 10% higher than that of a single airfoil. This optimal tandem arrangement modified the flow separation and the wake size, leading to enhanced lift in cases where the wake vortices are formed closer to the models.

It is important to note that previous research has left some unsolved questions that need further investigation. Firstly, the relative positions of the anterior and posterior bodies have not been thoroughly examined. In early studies by Miklasz et al. and Jafari et al., the posterior body was positioned below the anterior body. However, the 3D reconstruction of a flying snake gliding, illustrated in Figure 1b, and derived from actual footage of a snake gliding [15], shows that the posterior bodies can be elevated higher than the anterior body. This vertical configuration is attributed to the vertical bending associated with the undulation motion. According to the model proposed by Yeaton et al. [15], vertical bending consists of a sinusoidal wave (referred to as a “vertical wave”) and an up-and-down motion of the posterior body (known as “dorsal-ventral bending” [23]). As a result of these motions, various arrangements of tandem bodies can be observed. We presented a perspective view of a moment when the snake was performing aerial gliding. We selected three vertical planes parallel to the snake’s flight direction, as shown in Figure 1b. In the red and light gray planes, the posterior body is positioned slightly below the anterior part. However, in the blue plane, three cross-sections co-exist simultaneously, with the middle cross-section sitting above the anterior cross-section. This observation indicates that, in some cases, the posterior body is positioned higher than the anterior body, which has yet to be thoroughly investigated. Secondly, in Figure 1c, we present a series of time frames of the light gray vertical plane as seen from the side view. The anterior body stays still while the posterior body exhibits dynamic movement with a pitching down motion. This illustrates the dorsal–ventral bending motion, although the underlying flow physics of this phenomenon is still not fully understood. Preliminary results on the dynamic motion of the posterior body were discussed by Gong et al. [24], who focused on heaving motion. However, the impact of pitching motion, which affects the angle of attack of the posterior body, has not been examined.

The objective of this study is to analyze the aerodynamic interactions between upstream and downstream body segments, covering a wide range of spatial arrangements and accounting for dynamic motions. We used an in-house immersed boundary method (IBM) flow solver with a tree topological local mesh refinement (TLMR) technique to simulate a two-dimensional (2D) two-foil system, which effectively represents the anterior and posterior sections of a flying snake’s body. We quantified the aerodynamic performances of the foils, including lift and drag coefficients, as well as the lift-to-drag ratio. A detailed analysis of the wake structure and the vortex interactions between the upstream and downstream foils was performed. We also performed a parametric study on various combinations of relative distances. Additionally, we investigated the effects of different pitching motions, including various pitching frequencies and pitching angles. Lastly, the effects of the Reynolds number on the foil wake are examined. The remaining sections of this paper are organized in the following way. Section 2 details the simulation setup, including the snake-inspired foil shapes and the motion of the trailing foil in the computational domain (Section 2.1), and relevant information about the direct numerical simulation (DNS) method (Section 2.2). Section 3 focuses on the simulation results and our analysis. It is further broken into subsections describing the aerodynamics and performance of a solitary snake foil (Section 3.1), the static two-foil interaction at varying spacings (Section 3.2), the effect of changing Reynolds number (Section 3.3), and the effect of the pitching motion of the posterior foil (Section 3.4).

## 2. Materials and Methods

### 2.1. Problem Definition

**Figure 2:**
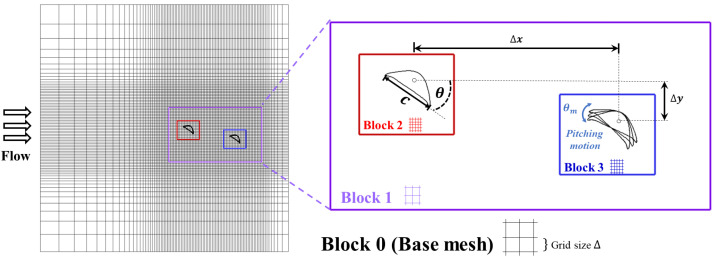
Schematic of the tandem snake airfoils mesh and setup of parameters. The base mesh has the smallest grid size of Δ = 0.0154c. The far field of the foils is surrounded by TLMR block 1, and the near field of the foils is surrounded by TLMR block 2 and 3. Each layer of blocks will double the density of the grid so that a higher resolution can be reached. In the zoomed-in view, the setup of the parameters is also shown, including the angle of attack (*AOA*) θ, the chord length *c*, and the horizontal and vertical distances between the centers of the foils Δx and Δy.

Many species in nature have developed appendages with unique shapes that grant them effective fluid dynamics, such as the flippers and flukes of cetaceans [26,27], the membranous wings of dragonflies [28], and, in this case, the cross-section shape of the flying snake [22,25]. The 2D cross-sections of a flying snake inspire the airfoil shapes used in the simulations in this study. During gliding, the flying snake would flex its rib cage around the spine to flatten out its ventral surface while the dorsal surface is still arched [20,21,22,25]. The chord length (*c*) is normalized to be used as a length unit. In the simulations conducted in the current study, we used semi-circular airfoil shapes with a round top surface and a flat bottom surface to emulate the cross-section of the flying snake, as shown in Figure 1c and Figure 2. Similar cross-sectional shapes have been observed in experiments [25] and used in prior computational works [20,21,22]. The setup of the immersed boundary method (IBM) simulations is shown in Figure 2. The two snake-inspired airfoils are placed in a tandem formation, with a horizontal spacing of Δx (the gap distance) and a vertical spacing of Δy (the stagger distance). Each foil is pitched at an angle of θ, which corresponds to the geometric angle of attack (*AOA*) of a foil. The real flying snake’s body during gliding would have a positive angle of attack [25]. In our simulations, we also kept the snake-like airfoils at a positive *AOA*. For the downstream foil, we also introduced a dynamic pitching motion $\theta_{m}$ to mimic the real motion of a flying snake. Previous computational works on solitary snake-inspired airfoils have found the optimal *AOA* to be 35° [20,21]. We reached the same finding in our single-foil study (Section 3.1) and used an *AOA* 35° in our two-foil setup for all the static cases. The snake airfoil downstream has relative positions.

For a starting case, we study the parametric pair (Δx, Δy) with the range of 3c≤Δx≤4c and −2c≤Δy≤2c at the increment of Δ=0.25c. Positive Δy is observed near the body’s head portion (see Figure 1b), which was not covered in the previous study. Furthermore, pitching is applied to the downstream airfoil for the dynamic motion study. The equation to describe the pitching is

(1) $$\theta =\theta_{0}+\theta_{m}sin\left(2\pi ft+\phi \right)$$

where $\theta_{0}$ is the *AOA* = 35° in the current study, $\theta_{m}$ and *f* represent the pitching amplitude and frequency, and ϕ is the pitching motion phase.

### 2.2. Numerical Method

The governing equations for the DNS solver are the incompressible Navier–Stokes equations given in Equations (2) and (3), where $u_{i}$ is the velocity components, *p* is the pressure, and *Re* is the Reynolds number:

(2) $$\frac{\partial u_{i}}{\partial x_{i}}=0;$$

(3) $$\frac{\partial u_{i}}{\partial t}+\frac{\partial (u_{i}u_{j})}{\partial x_{j}}=-\frac{\partial p}{\partial x_{i}}+\frac{1}{Re}\frac{\partial^{2}u_{i}}{\partial x_{j}\partial x_{j}};$$

The in-house DNS solver [29,30] employed in this study utilizes an immersed boundary method and finite difference algorithm to solve the governing equations. It has been extensively described in previous studies and validated across various scenarios [30,31]. This solver has been successfully applied to study flying snake aerodynamics [23,32,33] as well as canonical foil–foil interactions [31].

In the numerical solver, the equations are non-dimensionalized with the appropriate length and velocity scales. In this study, the non-dimensional parameter of the Reynolds number Re is defined as in Equation (4), where ν denotes the kinematic viscosity of the flow and $U_{\infty }$, and *c* holds the previous definition of the incoming flow and airfoil characteristic length. In the simulation, the initial Reynolds number is set up to be 1000, and the Reynolds number effect study will be held in a later discussion:

(4) $$Re=\frac{U_{\infty }c}{\nu }$$

Meanwhile, for the flow solver, the non-dimensional time $t^{*}$ is also defined by normalizing the computational time *t* with the following definition:

(5) $$t^{*}=\frac{tU_{\infty }}{c}$$

To quantify the aerodynamic performance, we calculate the lift and drag coefficients ($C_{L},C_{D}$) on each airfoil with the following definitions, where $F_{L}$ and $F_{D}$ represent the lift (perpendicular to the flow direction) and drag force (parallel with the flow direction):

(6) $$C_{L}=\frac{F_{L}}{0.5\rho U_{\infty }^{2}c},C_{D}=\frac{F_{D}}{0.5\rho U_{\infty }^{2}c}$$

Whereas lift and drag are commonly analyzed separately when discussing aerodynamic characteristics in flying and gliding, the lift-to-drag ratio (L/D) is generally employed to demonstrate the mechanical energy efficiency of the flight. In our study, the L/D is defined as the following:

(7) $$L/D=\frac{C_{L}}{C_{D}}$$

### 2.3. Simulation Setup

For all the 2D flow simulations, similar to the setup used in Ref. [21], a constant velocity incoming flow $U_{\infty }$ boundary condition is given at the inflow boundary. A zero-gradient velocity boundary condition is employed at the outflow boundary and the stream-wise velocity equal to the incoming flow is assigned to the top and bottom boundaries in order to let the vortices convect out of the border. Homogeneous Neumann boundary conditions are used for the pressure at all boundaries.

In the current solver, we improved the resolution of the grid near the foil by using the tree topological local mesh refinement (TLMR) technique [30]. Several layers of nested blocks were applied near the foil, and each layer of block creates a refined zone that subdivides the background mesh by a factor of 2 (twice as dense as its background mesh). The base mesh had a total count of half a million, while each nested refinement block had a similar grid count. The mesh density of the base layer had a grid size of Δ=0.0154c while blocks 2 and 3 had a grid size of Δ=0.00385c, achieving 260 grids per chord length. Following a similar computational setup [21], the incoming flow speed $u_{\infty }$ is chosen as 1 $c/t^{*}$ and the time step size Δt is chosen to be 0.001 $t^{*}$. This method with the TLMR technique enables the mesh refinement limited to the neighboring region, and thus the computation resources have been saved while keeping the resolution of the force computation and vortex structure on and around the computational models high.

A 20c × 20c non-uniform Cartesian grid is employed for the whole flow domain. The TLMR technique to improve the mesh density is applied to the mesh setup. Two layers of blocks are arranged in the nested blocks around the near field and the foils, the refined zone with size 6c × 4c is used to capture detailed wake structures, and around the upstream and downstream airfoil, a dense region with size 1.5c × 1.5c is deployed to resolve the boundary of the foil and calculate the force production accurately.

**Figure 3:**
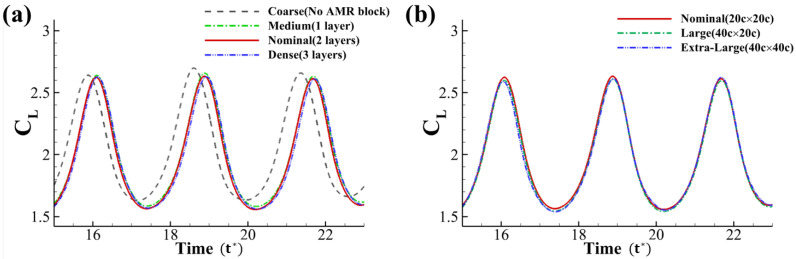
Grid and domain independence study. (**a**) Comparison of the lift coefficient of a solitary snake foil at *AOA* = 35° and *Re* = 1000 with four different grid densities. Coarse mesh applies only base mesh (block 0). Each layer of blocks will halve the grid size and double the grid density. (**b**) Lift coefficient of a solitary snake foil with three different domain sizes with the same mesh density. The nominal grid and domain are picked for all simulations.The time step for each time unit is 1000 steps.

We conducted the grid- and domain-independent study using a solitary foil at *AOA* = 35° and *Re* = 1000. Figure 3a compares the instantaneous lift coefficients of the pitching foil calculated on four different grid densities. Based on the TLMR technique, by adding one more layer of block near the foil, the densest grid will be halved in size, thus doubling the mesh resolution. We generated the coarse, medium, nominal, and dense mesh by adding the base mesh, one layer, two layers, and three layers of TLMR blocks near the foil. The result shows that while the coarse and medium mesh shows a large difference in lift performance, the difference in both the peak values and the average values between the nominal and dense grid was reduced to less than 0.2%. Thus, the nominal density of the mesh was applied for all simulations.

Furthermore, simulations were run with three different domain sizes with the same flow condition and mesh resolution around the pitching foil. As presented in Figure 3b, compared to the nominal domain used, the large 40c × 20c and extra-large 40c × 40c domains lead to a less than 1.0% difference in both the lift coefficient peak and time-averaged value. Thus, the grid dependence and domain dependence are precluded, and the nominal grid (setup shown in Figure 2) and domain here are chosen for all the 2D simulations.

## 3. Results and Discussion

In this section, we first examine the aerodynamic performance of a solitary foil at varying *AOA*s (Section 3.1) and cross-validate our result with that of Krishnan et al. [21]. The *AOA* that produces the best lift performance is used in the subsequent two-foil setup. As the wake produced by the upstream foil strongly affects the aerodynamics of the downstream foil, the vortex shedding and wake momentum are analyzed for the *AOA* = 35° case. Then, the aerodynamic performance and interactions in the two-foil system are analyzed in Section 3.2 with the configuration range of 3c≤Δx≤4c and −2c≤Δy≤2c. In Section 3.3, the effect of the Reynolds number is investigated by examining the same set of spatial arrangements at Re=500 and then focusing on one spatial arrangement of particular interest and varying its Reynolds number in a wider range. Finally, the effects of the pitching amplitudes and frequencies of the posterior foil are discussed in Section 3.4.

### 3.1. Solitary Foil: Aerodynamic Performance and Wake Dynamics

**Figure 4:**
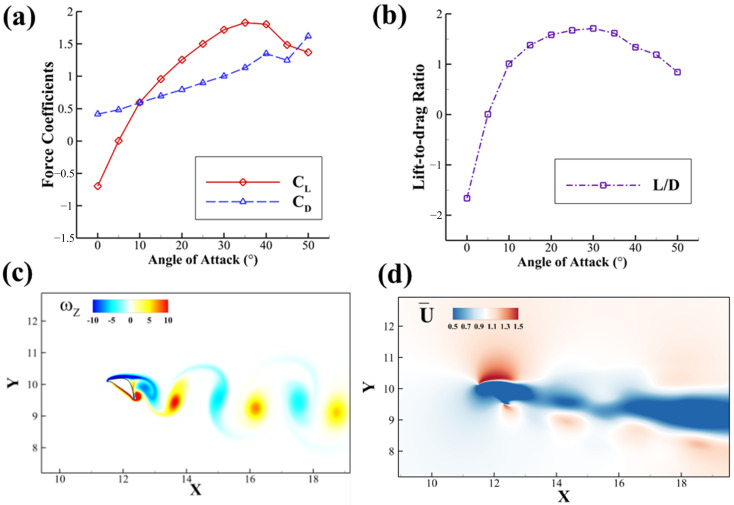
(**a**) Time-averaged lift coefficient $C_{L}$, drag coefficient $C_{D}$ with different *AOA*s, with *Re* = 1000; (**b**) lift-to-drag ratio L/D of a single flying snake airfoil at different *AOA*s; (**c**) vorticity contour for case *AOA* = 35°, *Re* = 1000 at peak lift production; (**d**) time-averaged stream-wise velocity field for the same case.

Our current study presents the aerodynamic performance of a solitary flying snake airfoil at different *AOA*s at *Re* = 1000 (Figure 4). This simulation is comparable with the 2D simulation conducted by Krishnan et al. [21]. The *AOA* ranges from 10° to 45° with an increment of 5°. The lift coefficient ($C_{L}$) increases vastly before 30° and reaches a peak value of 1.998 at 35°, which is the *AOA* we choose for further tandem foil study. Stall happens right after this critical *AOA*, where the $C_{L}$ decreases. On the other hand, the drag coefficient ($C_{D}$) increases linearly for nearly the whole *AOA* range. The lift-to-drag ratio L/D follows a similar convex trend that is observed in the $C_{L}$ trend and reaches a maximum at *AOA* = 30° with the value of L/D = 1.674.

As shown in Figure 4c, when the lift force is maximized at *AOA* = 35°, the foil produces a von Kármán vortex street with positive and negative vortices shed at the trailing edge of the snake airfoil. In Figure 4d, corresponding to the von Kármán vortex street, the average stream-wise velocity in the wake of the foil is reduced from the freestream, constituting a drag-type wake. The wake’s downward deviation shows the direction of vortex propagation and corresponds to vertical force production. The reverse flow in the blue region and the vortex wake can both influence the downstream airfoil’s aerodynamic performance.

### 3.2. Two Tandem Foil System: Aerodynamic Performance and Wake Dynamics

In this section, two flying snake airfoils are placed in tandem configuration. We investigate the aerodynamic performance and vortex structures based on different stream-wise and vertical distances between the foils. We start with the Reynolds number 1000 and *AOA* = 35°.

#### 3.2.1. Aerodynamic Performance

**Figure 5:**
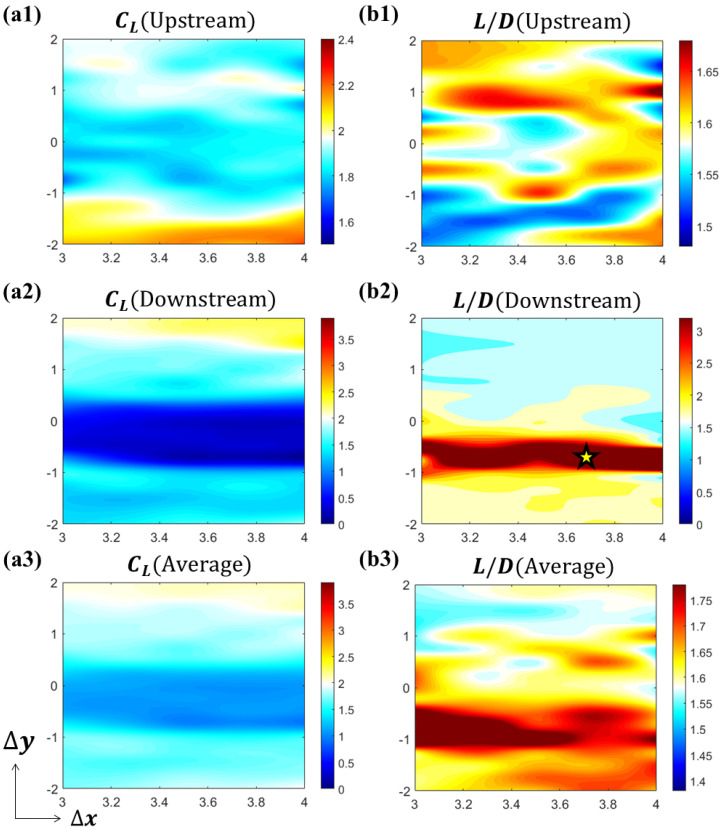
Aerodynamic performance of two tandem flying snake airfoils at *Re* = 1000. (**a1**–**a3**) time-averaged lift coefficients for upstream foil, downstream foil, and system average; (**b1**–**b3**) lift-to-drag ratio for upstream foil, downstream foil, and system average.

The aerodynamic performances of the two foils are summarized in Figure 5. The intermediate white region of the colormap is set to be the average value of the solitary foil ($\bar{C_{L}}$ = 1.9502, $\bar{L⁄D}$ = 1.5800). The hot region (red and yellow) represents performance enhancement, while the cold region (cyan and blue) represents performance reduction. From Figure 5a1–a3, it is shown that the interaction between the two foils reduces lift production in most cases. The lift production of upstream foil will be increased when the downstream foil is in the lower vertical position ($C_{L\_upstream}$ [Δx = 4.00c, Δy = −2.00c] = 2.2453), while the lift production of the downstream foil will be increased when itself is in upper vertical position ($C_{L\_downstream}$[Δx = 4.00c, Δy = 2.00c] = 2.3542). Figure 5a2 shows that the lift production of the downstream foil experiences a decrease when it is in the upstream foil’s wake, within the range of −1c≤Δy≤0.5c. However, in Figure 5b2, it is shown that the high L/D region for the downstream foil is also located in the upstream foil’s downstream, yet with a smaller range of −1c≤Δy≤−0.25c. This trend is also affecting the average system performance. We calculate the system average with the following:

(8) $$C_{L\_avg}=\frac{C_{L\_upstream}+C_{L\_downstream}}{2}$$

(9) $${L/D}_{avg}=\frac{C_{L\_avg}}{C_{D\_avg}}$$

Since the L/D performance is higher in downstream foil, the overall performance increases for the system average. A high L/D region shown in Figure 5b3 appears in a similar region as the downstream foil. The performance results show that although the two-foil system experiences a reduction in average lift, the lift-to-drag ratio generally benefits from the interaction between the foils within a wide range of relative positions. The performance (L/D) of the downstream foil especially benefits from the interaction with the upstream foil’s wake, especially within a band of −1c≤Δy≤−0.25c.

In the snake foil–foil interaction, the relative positioning and phase synchronization of the airfoils influence the aerodynamic performance, with wake capture mechanisms playing an important role. Similarly, the study by Tuncer and Platzer [34] demonstrates that in a tandem airfoil configuration, the presence of a stationary trailing airfoil in the wake of a flapping leading airfoil significantly enhances thrust and propulsive efficiency due to favorable wake interactions. While the tandem airfoil system in the study primarily emphasizes thrust augmentation, in the flying snake, the interaction between foils likely affects both lift and drag forces, impacting stability and maneuverability in gliding flight.

Numerous flying or gliding animals have been found to adjust their body shapes to enhance efficiency or to maneuver. The ancient feathered dinosaur *(Microraptor)* may have laid its legs flat to achieve a better L/D ratio for a longer horizontal gliding capability [35]. It is reported that flying fish would expand their pelvic fins when flying close to the surface of the sea to decrease the drag force and elevate the L/D [36]. Ornithologists have determined that birds can adjust their wing camber and aspect ratio through the practice of functional morphology, leading to a greater L/D [37]. The lift-to-drag ratio can be adversely influenced by factors such as alteration in the wing transmissivity and aeroelasticity, which influence the production of lift and drag [38], or the molting of feathers, which lowers the L/D ratio to 7 from 10.5 when the feathers are full [39]. Some birds are able to modify their lift-to-drag ratios with each wingbeat, allowing them to utilize aerodynamic force vectoring and maneuver up to an angle of approximately 100° relative to the horizontal plane [40]. In the current study, we may explore different parameters and find the effects on the L/D performance.

#### 3.2.2. Wake Structure

**Figure 6:**
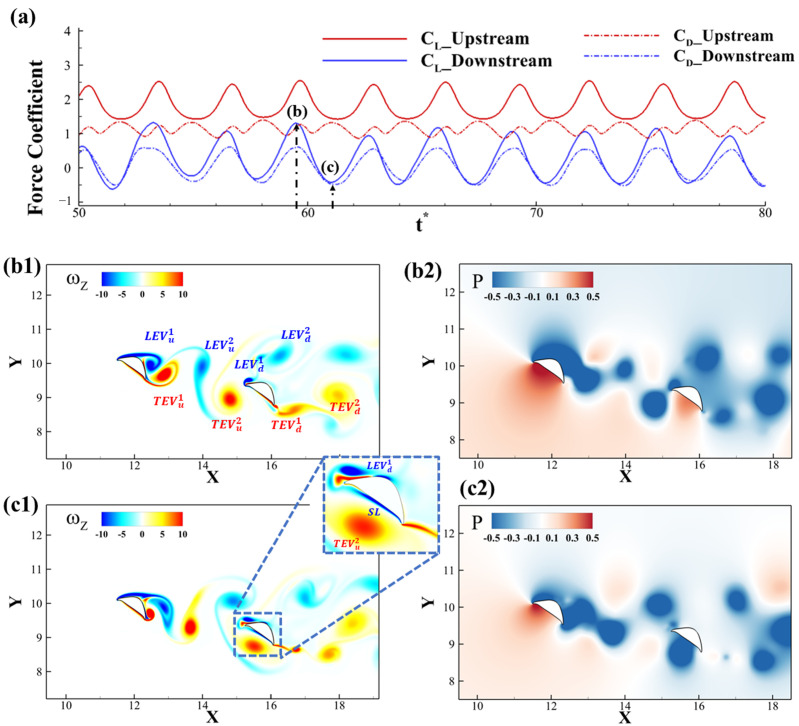
(**a**) instantaneous lift and drag coefficients of the two foils over time, (**b1**,**c1**) vorticity contours and (**b2**,**c2**) pressure contours for the configuration Δx = 3.75c, Δy = −0.75c at the instants of the maximum lift production on downstream foil (**b1**,**b2**), and the minimum lift production (**c1**,**c2**).

In Figure 6, the force history for two foils as well as the flow information for the best-performing configuration (Δx = 3.75c, Δy = −0.75c) is presented. This configuration is shown in Figure 6 with a star as it has the best L/D for downstream foil. Compared with solitary foil, it is clearly observed that the force production is reduced for the downstream foil, as shown in Figure 6a. We will pick the peak and trough lift production frame for further explanation about the vortex formation. As shown in Figure 6b1, the upstream foil sheds $TEV_{U}^{1}$ from the trailing edge and it propagates downstream. Meanwhile, the $LEV_{D}^{1}$ and $TEV_{D}^{1}$ formed on the edges provide two low-pressure regions, which are shown in Figure 6b2. With the vortex $TEV_{U}^{1}$ traveling downstream, it will reach the ventral surface of the downstream foil, inducing a negative shear layer, as shown in Figure 6c1(inset). As shown in Figure 6c2, the strong rotation of the shed TEV from the upstream foil creates a strong negative pressure region on the ventral surface of the downstream foil, explaining the low lift production at this instant. The low lift production does not necessarily mean a low lift-to-drag ratio performance since its drag is also small due to the forward-facing ventral surface of the downstream foil.

**Figure 7:**
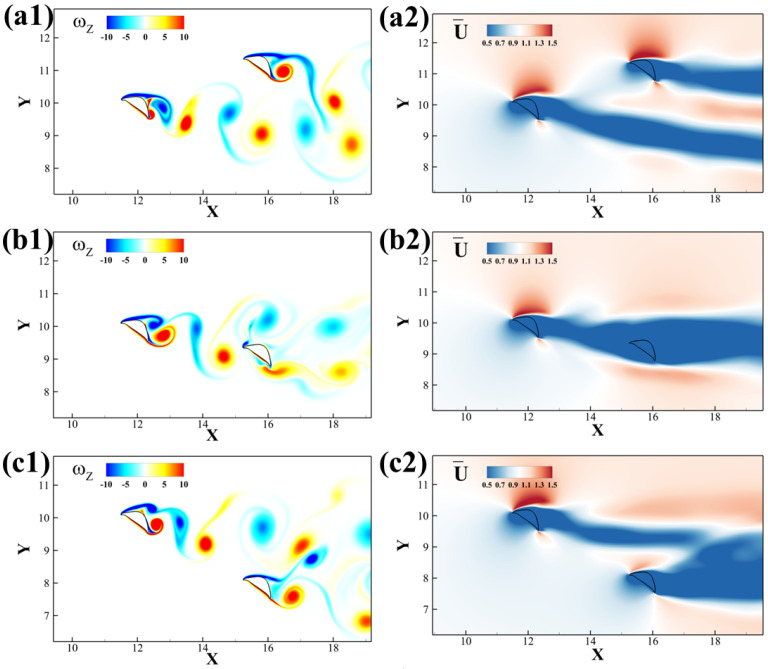
(**a1**–**c1**) vortex contours and (**a2**–**c2**) time-averaged stream-wise velocity fields of two tandem flying snake airfoils under different configurations: (**a**) Δx = 3.75c, Δy = 1.25c, (**b**) Δx = 3.75c, Δy = −0.75c, (**c**) Δx = 3.75c, Δy = −2.00c.

The time-averaged stream-wise velocity contour is presented in Figure 7 to investigate the relationship between different configurations. The upstream foil produces a drag-type von Kármán vortex wake, as shown in Figure 7a1,b1,c1, and correspondingly, the streamwise momentum in the wake of the upstream foil is much less than that of the free stream, as shown in Figure 7a2,b2,c2. As a result of the lift force production on the anterior foil, its wake exhibits a slight downward deflection. The cases with L/D ratios better than a solitary foil generally have the posterior foil inside the wake of the upstream airfoil. By taking advantage of the oncoming vortex wake, the downstream foil almost reaches zero drag, leading to a high L/D performance, as demonstrated in Figure 7b1,b2. Cases with L/D less than that of a solitary foil, such as shown in Figure 7a1,a2, have the posterior foil outside of the drag wake of the upstream foil, so not only is it encountering the full momentum of the free stream, but its lift production is also interrupted by the incoming vortex flow and the oscillating flow pressure.

**Figure 8:**
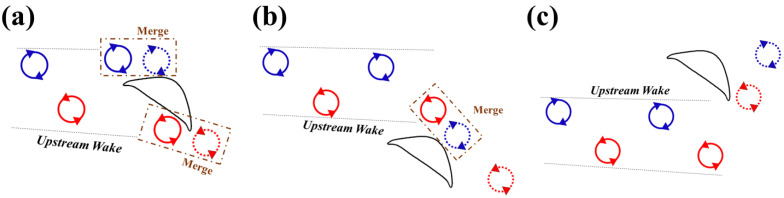
Schematic of vortex interaction when the downstream foil is located (**a**) in the middle of (**b**) below (**c**) above the upstream wake. Vortices originating from the upstream and downstream foils are depicted in solid and dashed lines, respectively.

Figure 8 summarizes the primary vortex structure for different configurations of the foils. As previously mentioned, the upstream wake is a typical von Kármán vortex street. When the downstream foil is positioned in the center of the wake, both the leading-edge vortex (*LEV*) and trailing-edge vortex (*TEV*) combine with vortices generated by the upstream foil. If the downstream foil is placed below the wake, the *LEV* on its upper surface merges with the *TEV* from upstream. Conversely, when the downstream foil is situated above the wake, the *TEV* on its lower surface interacts with the upstream *LEV*.

The wake information also explains the lift production in Figure 5. The high lift production for upstream and downstream foils are found in Δy≤−1.5c and Δy≥1.5c, respectively. Those are the positions where the wakes are separated. When the upstream foil is higher, it will experience the interaction and the force lifting it while the downstream foil’s lift reduces. The upper foil experiences a slightly higher lift while the lower foil experiences the counter force and reduces the lift. The same explanation holds when the upstream foil is lower and the downstream foil has a better overall performance. Nevertheless, when the wake merges into one, the downstream foil takes advantage of the wake and brings a higher performance in the lift-to-drag ratio, leading to higher gliding efficiency.

### 3.3. Effects of Reynolds Number

In this section, we focus on the effects of the Reynolds number on aerodynamic performance and vortex dynamics.

#### 3.3.1. Aerodynamic Performance

**Figure 9:**
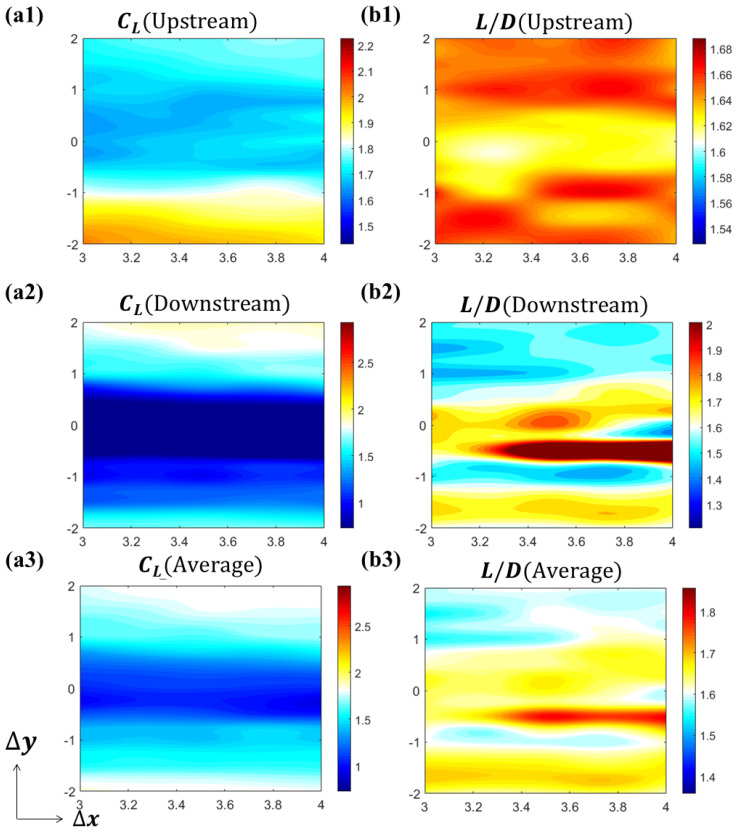
Aerodynamic performance of two tandem flying snake airfoils at *Re* = 500. (**a1**–**a3**) time-averaged lift coefficients for upstream foil, downstream foil, and system average; (**b1**–**b3**) lift-to-drag ratio for upstream foil, downstream foil, and system average.

The two-foil study is conducted at varying Reynolds number values. Figure 9 shows the aerodynamic performance of the two foils in varying spatial arrangements given Re=500.

Generally speaking, *Re* = 500 and *Re* = 1000 share a similar pattern in aerodynamic performance. When downstream foil is located in Δy≤−1.5c, the lift production for upstream foil outperforms the solitary foil. But, the lift improvement for the downstream foil occurs for Δy>−1.5c, and the lift production for downstream foil is better than the solitary foil. The high L/D region for the downstream foil is still located in the wake of the upstream foil. By calculating the cycle average, it is shown in Figure 9a3 that the lift production of the system is reduced due to the interaction. The lift-to-drag ratio, on the other hand, is raised when the downstream foil is located in the wake of the upstream foil. However, there are some differences that need to be noted. First, the average value of solitary foil is now $\bar{C_{L}}$ = 1.8276, $\bar{L⁄D}$ = 1.6078, which shows a similar trend as previous Reynolds number effect studies [20,21]. Second, the high L/D region’s width reduces, indicating that the strength of the wake fades. Also, the hot region shifts upward compared with the higher Reynolds number case, indicating a deviation in the wake due to changes in the Reynolds number, which will be addressed in the upcoming section.

#### 3.3.2. Vortex Dynamics

In this section, we made a comparison between different Reynolds number cases to provide a better understanding of vortex dynamics and its relation to aerodynamic performance. We kept the exact configuration of Δx = 3.75c, Δy = −0.75c and altered the Reynolds number.

**Figure 10:**
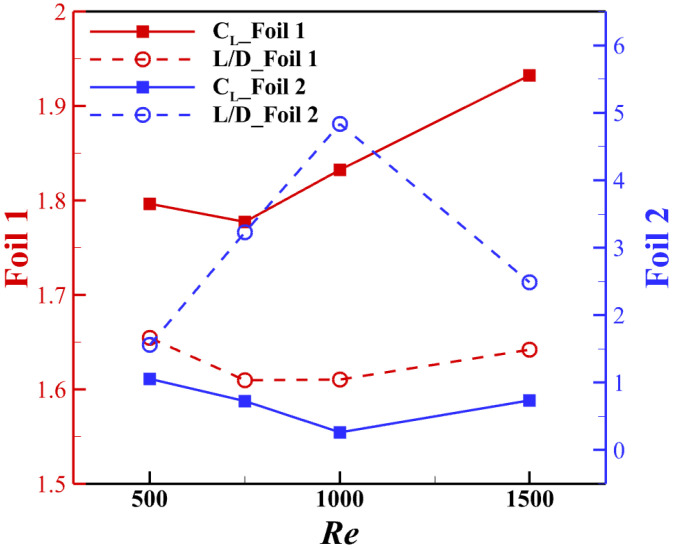
Aerodynamic performance of the two foils in the configuration Δx = 3.75c, Δy = −0.75c given different Reynolds numbers.

From the performance contour presented in Figure 5 and Figure 9, it can be predicted that with the Reynolds number increase, the lift production reduces in the wake region, yet the lift-to-drag ratio increases. Also, the wake region will shift slightly with the Reynolds number increase. The aerodynamic performance variation due to changes in the Reynolds number in this spatial configuration is presented in Figure 10. From the results, it can be seen that for the best lift-to-drag ratio configuration at *Re* = 1000, either increasing or decreasing the Reynolds number will affect the performance of the downstream snake foil, and the lift-to-drag ratio is affected more than the lift force. In summary, by adjusting the Reynolds number, the high efficiency (high L/D) region will shift downward when the Reynolds number increases. This further suggests that the vortex shedding direction may change and that a slight wake deviation occurs. We examine the reason behind this by analyzing the wake and vortex dynamics.

**Figure 11:**
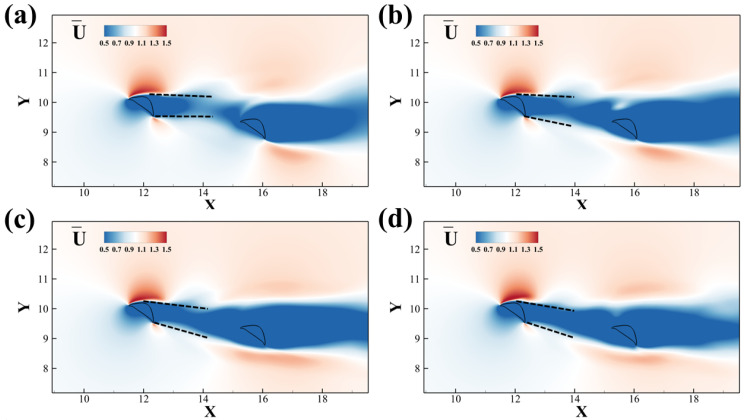
Time-averaged stream-wise velocity fields of two tandem flying snake airfoils under different Reynolds numbers: (**a**) *Re* = 500, (**b**) *Re* = 750, (**c**) *Re* = 1000, (**d**) *Re* = 1500.

We present the time-averaged velocity fields in Figure 11. The deviation angles of the wake from the upstream foil increase with the Reynolds number, which is indicated by the dashed line. A higher deviation angle corresponds with the lift production increase for upstream foil. The performance of the downstream airfoil is also highly related to the wake and vortex interaction, yet less explicit.

**Figure 12:**
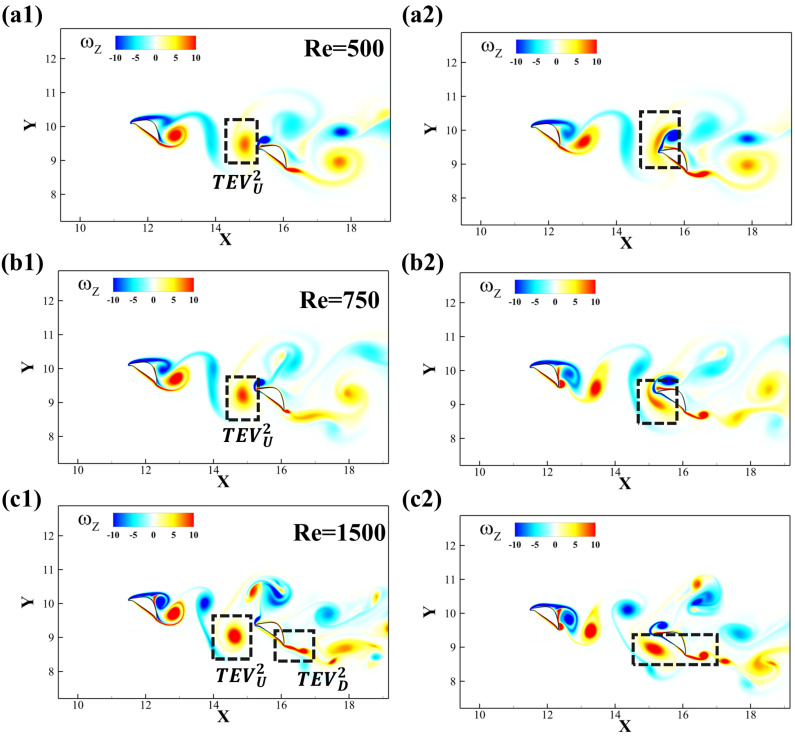
Vorticity contour for the configuration Δx = 3.75c, Δy = −0.75c at *Re* = 500, 750 and 1500 for (**a**–**c**). (**a1,b1,c1**) and (**a2,b2,c2**) represent the instants of the maximum and minimum lift production on foil 2, respectively.

To further investigate the formation and interaction of the vortices near the downstream snake foil, the instantaneous vorticity contours at maximum lift production for different Reynolds number cases are presented in Figure 12. In Figure 12a1,b1,c1, the $TEV_{U}^{2}$ meets the leading edge of the downstream airfoil. Different Reynolds numbers have slightly different angles of incidence and initial position, leading to different vortex–wedge interaction patterns. In Figure 12a2, the $TEV_{U}^{2}$ collides with the wedge (leading edge) of the downstream foil, inducing the shedding of the *LEV* of the downstream foil, and then travels with it as a vortex pair over the dorsal surface. Tucker et al.’s work [41] provided similar vortex–wedge interaction profiles. By shifting the initial position of the vortex downward with the increasing Reynolds number, the vortex breakdown switches from the dorsal surface to the ventral surface, which happens in Figure 12b2 at *Re* = 750. As a result, the $TEV_{U}^{2}$ distorts and merges with the shear layer on the ventral surface. At a higher Reynolds number *Re* = 1500, the $TEV_{U}^{2}$ advects downstream without breaking down at the wedge. Figure 12c2 shows that it maintains its shape before merging and interacting with the $TEV_{D}$ generated at the trailing edge of the downstream foil. Similar trends of position shift along with vortex breakdown have been summarized in Refs. [42,43].

### 3.4. Effects of the Dynamic Motion

In this section, the effects of the dynamic motion of the downstream snake airfoil on the system’s hydrodynamic performance and wake evolution are investigated. As described in the methodology section, dynamic pitching is applied to the downstream foil according to Equation (1). Here, we investigate the effect of varying pitching amplitude and pitching frequency.

#### 3.4.1. Effect of Pitching Amplitude

**Figure 13:**
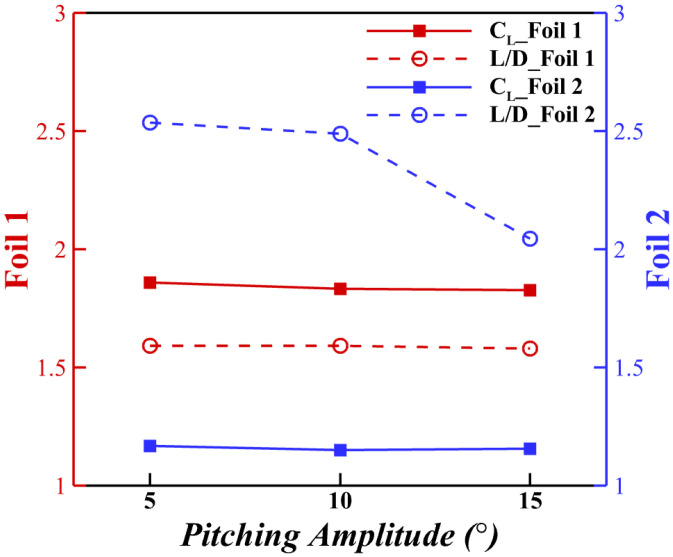
Aerodynamic performance of different pitching amplitudes at configuration Δx = 3.75c, Δy = −0.75c.

The pitching amplitude determines the angle of attack range of the trailing foil. As shown in Equation (1), the *AOA* for posterior airfoil can vary cyclically. The lift and drag force coefficients of varying pitching amplitudes are presented in Figure 13. As the pitching amplitude increased, the lift production on both foils decreased due to the onset of stall. This holds for the optimal $AOA=35^{\circ }$; either increasing (pitch up) or decreasing (pitch down) the *AOA* will worsen the lift production.

**Figure 14:**
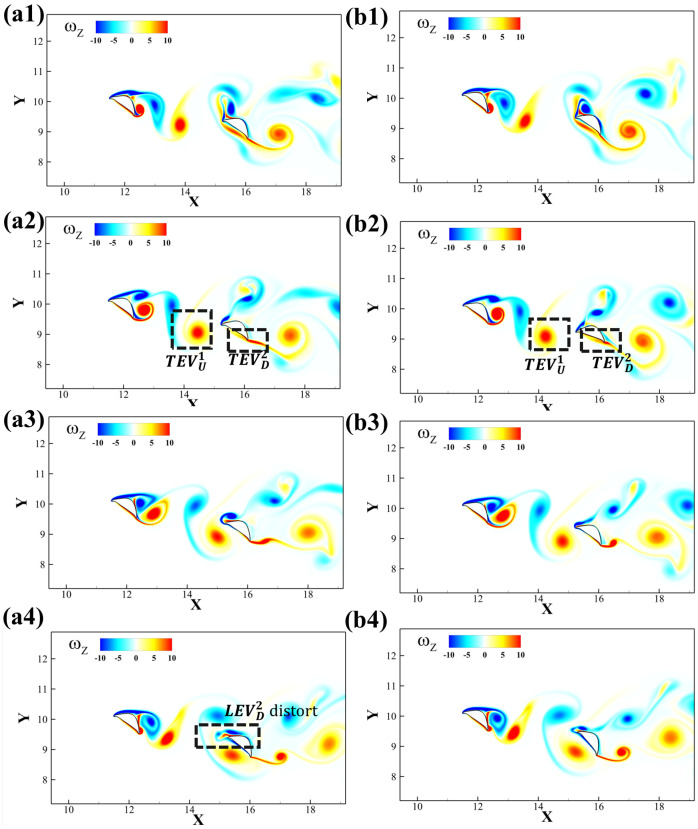
Evolutions of the vortex structures in one pitching period for the pitching amplitude 5° (**a1**–**a4**) and 15° (**b1**–**b4**). Each time frame represents 1/4 of the pitching period.

**Figure 15:**
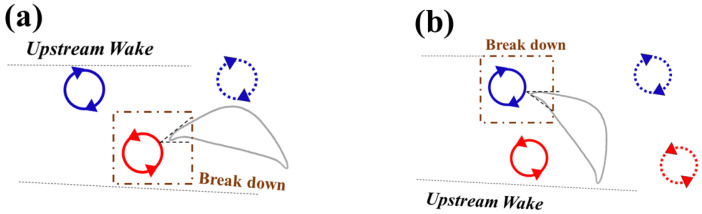
Schematic of vortex interaction when the downstream foil is at (**a**) minimum and (**b**) maximum pitching amplitude.

The vortex interaction becomes more complicated due to the introduction of the posterior foil’s pitching motion. A time sequence of the vortex shedding and capture is shown in Figure 14. The introduction of the dynamic pitching leads to the dynamic variation in the *AOA* on the downstream foil. The mean *AOA* = 35° provides maximum lift as well as the transition of the vortex generation [21]. As the geometric angle of attack of the downstream foil continues changing, so does the incident angle of the foil wedge relative to the upstream wake. Figure 15 presents the vortex formation process at different amplitudes 5° and 15°. When the amplitude is small, the leading edge of the downstream foil collides with the upstream *TEV* while when the amplitude is large, as its leading edge would collide with the upstream *LEV*.

In Figure 14a2,b2, the $TEV_{U}^{1}$ advects downstream to the ventral surface of the downstream foil. Due to different amplitudes, $TEV_{D}^{2}$ at a low amplitude breaks down into a thin layer, yet a high amplitude forms a compact vortex. Also, in Figure 14a4,b4, the $LEV_{D}^{2}$ merges with $LEV_{U}^{2}$, yet the $LEV_{D}^{2}$ distorts and stretches in a low-amplitude case.

#### 3.4.2. Effect of Pitching Frequency

In this section, the pitching frequency of the downstream foil is changed. According to the following definition:

(10) $$T=\frac{1}{f}$$

we conducted the simulation using *T* = 1 ∼ 6. The time period also corresponds with the simulation time. The force history shown in Figure 3 shows that the solitary foil vortex shedding period is *T* = 3.2 using FFT. However, with the introduction of downstream foil, the period rises to *T* = 4 for the upstream foil. According to previous studies [24], phase change affects the interaction between tandem foils. Therefore, the frequency variation will affect the vortex formation and interaction accordingly.

**Figure 16:**
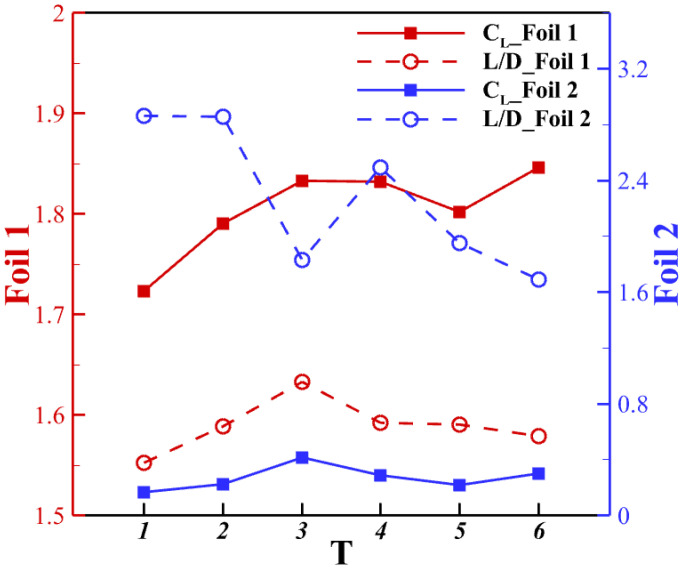
Aerodynamic performance of different pitching frequencies in the configuration Δx = 3.75c, Δy = −0.75c.

**Figure 17:**
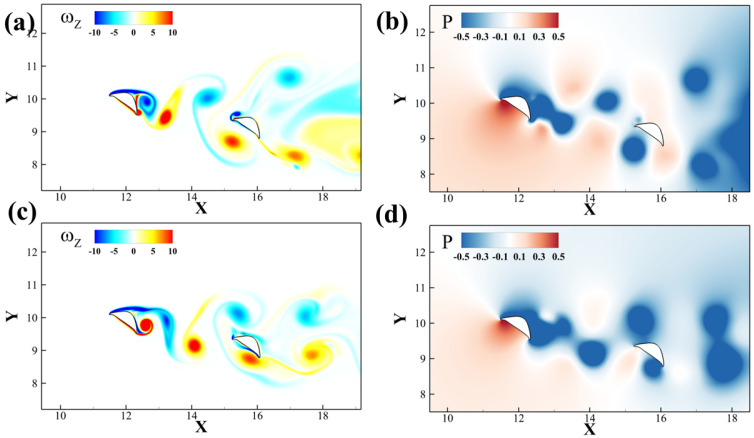
(**a**,**b**) show the instantaneous vorticity and pressure contour at *T* = 3. (**c**,**d**) show the same information for *T* = 5.

The time-averaged aerodynamic performance is presented in Figure 16. It is observed that for the upstream foils, the lift production and the lift-to-drag ratio will be affected by the interaction with the downstream airfoil, but only within 5 %. The downstream airfoil’s performance changes significantly with the pitching frequency. At *T* = 3, when the pitching motion is synchronized with vortex shedding from the upstream wake, it will provide a high lift production. Figure 17a shows the differences in vortex patterns for different frequencies, at their respective instants of peak lift production. Figure 17c shows a different pattern by comparing the same time frame of peak lift for downstream foil. In Figure 17b,d, the pressure contour near the upstream foil shows a similar pattern of strong negative pressure on the dorsal surface in both pitching frequencies, yet the pressure difference is higher at *T* = 3 since the upper surface does not have a low-pressure region. For the downstream foil, the pressure difference of the *T* = 3 is more considerable, thus leading to the 93% increase in lift production compared with *T* = 5.

The vortex interaction contributes not only to lift production but also to the lift-to-drag ratio. At *T* = 1, 2, and 4, the L/D ratio of the downstream foil reaches a higher level than others. The picked-out cases show integer multiples of pitching frequency, where the vortex interaction is synchronized with each other.

## 4. Conclusions

This paper presents a numerical simulation of a tandem airfoil model inspired by the body cross-section of a flying snake. A comprehensive investigation was conducted on the aerodynamic performance of both the upstream and downstream airfoils. We examined various parameters, including the relative position configuration, Reynolds number, and the effects of dynamic motion. Our detailed analysis revealed the structure of the vortices, which consists of the leading-edge vortex (*LEV*) and trailing-edge vortex (*TEV*). Additionally, we studied the interaction between the wakes of the tandem airfoils.

Compared to a solitary snake airfoil, the performance of a two-foil system is significantly influenced by the relative positions of the foils under all conditions considered. For the upstream foil, the changes in performance are relatively minor; it can either outperform the solitary foil in terms of lift production when the downstream foil is lower than the wake of the upstream foil, and the L/D of the upstream foil varies very little. In contrast, the downstream foil experienced a substantial decrease in lift when positioned in the wake of the upstream foil. However, this configuration leads to a big increase in the lift-to-drag ratio corresponding to greater gliding efficiency. Specifically, the downstream foil experiences a lift reduction of 86.5% and a drag reduction of 96.3%, resulting in a 377% increase in the lift-to-drag ratio compared to a single foil. Overall, the performance of the entire system reflects a similar trend to that of the downstream foil, as it exhibits much greater variation than the upstream foil.

The results from a flow field analysis indicated that the lift-to-drag ratio of the downstream foil is maximized when it is positioned in the wake of the upstream foil. The interaction between the vortices leads to a reduction in both lift and drag production, as the pressure difference between the upper and lower surfaces decreases significantly when the captured vortex breaks down and merges with the ventral surface. As a result, the posterior body of the snake sacrifices lift production in order to achieve greater efficiency while gliding.

Further research on the Reynolds number effects revealed that the flow structure remains similar as the Reynolds number increases. However, this change primarily affects the strength of the vortices and causes a deviation in the wake. As the Reynold number increases, the best-performing parametric region shifts downward (resulting in a decrease in Δy), necessitating a corresponding adjustment of the downstream foil to optimize its performance.

The performance of the two-foil system was enhanced with a downstream foil that undergoes dynamic pitching. This study also examines both the aerodynamic performance and vortex dynamics of such a system. The aerodynamic performance and the corresponding vortex wakes change with the change in the foil pitching amplitude and frequency due to the phase modification of vortex capture. As the shed vortex moves downstream toward the posterior foil, the motion of the foil leading edge alters the interaction pattern between the vortex and the wedge (the foil leading edge), which depends on the phase of the motion. When the vortex shedding aligns with the dynamic pitching motion, lift production increases through the introduction of movement at the appropriate amplitude and frequency. However, the L/D efficiency decreases at the cost of overall performance.

The two-dimensional model simplifies the complex three-dimensional motion of snake gliding and neglects critical effects such as lateral undulations, body torsion, and vortex structures, which may influence aerodynamic forces and stability. However, despite this limitation, this study provides valuable insights into the tandem airfoil gliding mechanisms of flying snakes, laying a foundation for optimizing gliding postures. Moreover, the simplified framework allows for the incorporation of posterior body dynamics observed in real snake gliding, making it a more canonical problem and enhancing our understanding of the two-foil system. This balance between simplification and insight contributes to both fundamental aerodynamics and the design of bio-inspired flying robots.

## Data Availability

The raw data supporting the conclusions of this article will be made available by the authors upon request.
